# The low risk for early renal damage during lithium treatment has not changed over time

**DOI:** 10.1177/02698811221123054

**Published:** 2022-09-19

**Authors:** Mihaela Golic, Harald Aiff, Per-Ola Attman, Bernd Ramsauer, Staffan Schön, Steinn Steingrimsson, Jan Svedlund

**Affiliations:** 1 Department of Psychiatry and Neurochemistry, Institute of Neuroscience and Physiology, Sahlgrenska Academy, University of Gothenburg, Göteborg, Sweden; 2 Department of Nephrology, Institute of Medicine, Sahlgrenska Academy, University of Gothenburg, Göteborg, Sweden; 3 Department of Nephrology, Skaraborg Hospital, Skövde, Sweden; 4 Swedish Renal Registry, Jönköping County Hospital, Jönköping, Sweden

**Keywords:** Lithium, adverse effects, chronic kidney disease, renal impairment, renal damage

## Abstract

**Background:**

Modern lithium management guidelines were introduced to improve the renal prognosis of lithium patients.

**Aims:**

To examine whether prospects for severe renal impairment (defined as chronic kidney disease at least stage 4 (CKD4)), in long-term lithium patients, have changed over time after the introduction of lithium monitoring guidelines.

**Methods:**

The time to and hazard for CKD4 were compared between three patient cohorts who started long-term lithium in three consecutive decades: 1980s, 1990s and 2000s. The follow-up time was 10 years after completion of 1-year treatment. The data were collected from Sahlgrenska University Hospital’s laboratory database.

**Results:**

In all, 2169 patients were included: 623 in Cohort 1 (started lithium during 1980s), 874 in Cohort 2 (1990s) and 672 in Cohort 3 (2000s). Compliance with lithium monitoring guidelines improved, and mean serum lithium decreased, through the cohorts. In all, 22 patients developed CKD4 during follow-up. The time to CKD4 was the same in all three cohorts (overall: 10.96 years, 95% confidence interval: 10.94–11 years). Age and serum creatinine concentration at start were significant risk factors, while sex had no prognostic value. After adjusting for the significant covariates, there was no statistically significant difference in the hazard for CKD4 between the three cohorts.

**Conclusion:**

The risk for severe renal damage during the first decade of long-term lithium is low, but has not changed over time. Our data suggest that improved compliance with lithium guidelines is not reflected in less risk for severe renal damage.

## Introduction

Lithium is the first choice for long-term treatment of bipolar disorder and is a valuable augmentation strategy for treatment-resistant unipolar depression. Lithium’s mood stabilising effect (Baldessarini et al., 2019) and anti-suicidal effect (Smith and Cipriani, 2017) have been repeatedly reported over a long period of time, while more recently, a neuroprotective effect has been postulated supported by preclinical, imaging and animal studies (Forlenza et al., 2014).

Side effects of treatment with lithium are also well known, most of them (such as hypothyroidism, hand tremor, polyuria) readily recognisable and potentially manageable. Long-term side effects such as severe deterioration of renal function are uncommon, but difficult to predict, prevent or manage (Davis et al., 2018). Early reports on irreversible kidney damage among lithium patients (Hestbech et al., 1977) have served as a starting point for developing new and stricter guidelines (Amdisen et al., 1980) for lithium management. The guidelines aimed to enable timely detection and management of severe kidney disease, hypothyroidism and hyperparathyroidism in lithium-treated patients.

Modern lithium management guidelines are considered to reflect best practice and were expected to improve the renal prognosis of lithium-treated patients. This presumption is also supported by the increased compliance with lithium treatment guidelines over time (Golic et al., 2018), as well as the general advances in the management of diabetes and cardiovascular diseases over the last decades (Deeb, 2008; Mensah et al., 2017), given the fact that chronic cardiovascular and renal morbidity coexist in the same patient and can aggravate each other (Thomas et al., 2008).

However, the question still remains – whether increased compliance with guidelines and general improvement in the clinical management of somatic comorbidity have indeed improved renal outcomes.

The aim of this study was to examine whether the risk for severe renal impairment (defined as chronic kidney disease at least stage 4 (CKD4)), in long-term lithium-treated patients, has changed over time, following the introduction of the new monitoring guidelines in Sweden.

It was hypothesised that the risk for serious renal impairment had decreased over time.

## Methods

### Patient selection

Patients were identified in the laboratory database at the Department of Clinical Chemistry at Sahlgrenska University Hospital, Gothenburg, Sweden as described in a previous publication (Aiff et al., 2015). For this study, date and values for serum lithium concentrations (*S-Li*) and serum creatinine concentrations (*S-Creatinine*) between 1980 and 2017 were retrieved, together with patients’ unique ID number, sex and birth date. Eligible patients were those who had one or more S-Li and one or more S-Creatinine between 1980 and 2009, and were 18 years or older at the first S-Li.

Patients who had at least one year of continuous lithium treatment (further referred to as *Index Treatment*) within the same calendar decade as their first S-Li were included in the study. Excluded were patients whose renal function could not be evaluated at the start of Index Treatment (*Index Start*) or at the last S-Li, patients with S-Creatinine above the reference interval at Index Start, as well as patients with less than one year of follow-up.

After applying the above inclusion and exclusion criteria, patients were grouped in three chronologically consecutive cohorts according to the date of Index Start: Cohort 1 included patients with Index Start between 1980 and 1989, Cohort 2 between 1990 and 1999 and Cohort 3 between 2000 and 2009. Patients were followed up to 10 years after Index Treatment.

### Measurements

The S-Li concentration was determined by flame photometry. S-Creatinine was measured by picrate method until 1 June 2004 and by an enzymatic method thereafter. The enzymatic method is more specific, and an adjustment of the values obtained before June 2004 was necessary, to be comparable to the later ones (Aiff et al., 2015). The reference interval for S-Creatinine is 45–90 μmol/l for women and 60–105 μmol/l for men.

Date of Index Start and corresponding S-Creatinine (*S-Creatinine at Index Start*) were identified for each patient. The glomerular filtration rate was estimated (eGFR) and calculated from the S-Creatinine, *Sex* and *Age*, according to the revised Lund–Malmö formula (Bjork et al., 2011; Swedish Council on Health Technology Assessment (SBU), 2013). Obvious erroneous S-Creatinine at Index Start values were ignored in favour of the next consecutive value.

*Time on Li*, *Mean S-Li* and *Compliance* with lithium monitoring guidelines were calculated for every patient, taking into consideration the active lithium treatment periods. Periods with at least one S-Li per year were regarded as active treatment periods. Periods of 1 year or more without any S-Li were regarded as treatment interruptions and were excluded from the computation of these variables. Thus, Time on Li was the sum of the active lithium treatment periods and the Mean S-Li was calculated dividing the area under the S-Li curve of the active treatment periods, by the Time on Li. Lithium monitoring guidelines require examination of S-Li and S-Creatinine at least every 6th month (American Psychiatric Association, 2010; Bendz and Aurell, 2004). Compliance was calculated as the proportion of Time on Li for which these basic monitoring requirements were fulfilled.

*Li Intoxications* were defined as episodes of S-Li 1.5 mmol/l and higher.

### Outcomes

The end point of interest (*Event*) was defined as reaching CKD4, according to the classification by Kidney Disease Outcomes Quality Initiative (National Kidney Foundation, 2002), that is, repeated eGFR values consistently below 30 ml/min/1.73 m^2^ for a minimum of 3 months.

*Survival Time* was defined as time from Index Start to either Event or end of follow-up.

Patients who received renal replacement therapy (RRT) were identified in the Swedish Renal Registry (2019).

### Statistical analysis

Patients’ characteristics and treatment-related variables were compared between the groups. The independent samples t-test was applied for comparing two consecutive means between decades. Mann–Whitney U test was used for small samples with non-normal distribution. For categorical variables, Pearson’s chi-square test was used.

A survival analysis was conducted, comparing the Survival Time between the three cohorts. Kaplan–Meier graphs were used to illustrate the Cummulative i﻿ncidence of Event (CKD4). The longest total follow-up time was 11 years (Index Treatment duration of 1 year plus 10 years after Index Treatment). Thus, right censoring occurred after 11 years of follow-up, at death or at loss to follow-up. The Log Rank test (Mantel–Cox) was used to compare the equality of Survival Times. In all analyses, a *p* value <0.05 was considered statistically significant. Cox proportional hazards model was used to look for association between decade of start and outcome and to adjust for possible covariates (Sex, *Age at Index Start* and *S-Creatinine at Index Start*). The null hypothesis was that Survival Time is the same for all three cohorts.

Microsoft Excel 2019 (Microsoft Corp, Redmond, WA, USA) and MatLab 2019a (MathWorks, Natick, MA, USA) were used for data processing. SPSS Statistics v. 28 (IBM Corp, Armonk, NY, USA) was used for statistical analyses.

## Ethical considerations

The study was approved by the Regional Ethical Review Board in Gothenburg and performed in accordance with the Declaration of Helsinki, as revised in 1989. The ethical review board approved the retrieval of laboratory data and subsequent chart reviews without written or verbal consent from patients.

## Results

### Flowchart

**Figure 1.**
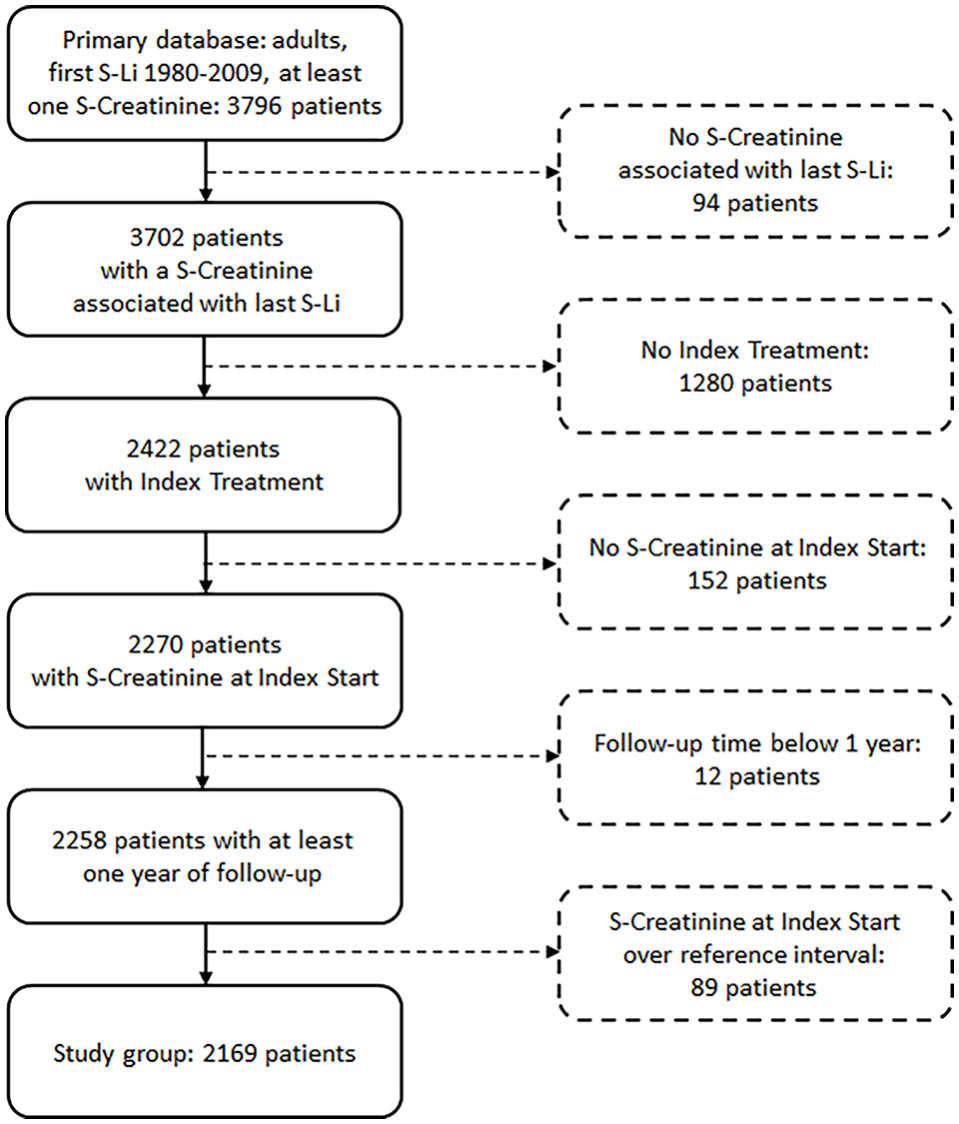
Study flowchart.

After applying the inclusion and exclusion criteria, a total of 2169 patients were included in the study (Figure 1).

### Characteristics of the cohorts

The start variables of the three cohorts are presented in Table 1, where all cohorts included more women than men, Age at Index Start was lower in the latest cohort and S-Creatinine at Index Start increased through the cohorts.

**Table 1:** Patients’ characteristics in each cohort.

	Cohort 1 (*N* = 623), 1980–1989	Cohort 2 (*N* = 874), 1990–1999	Cohort 3 (*N* = 672), 2000–2009
Sex			
Women *n* (%)	398 (64)	512 (59)^a^	406 (60)^b^
Men *n* (%)	225 (36)	362 (41)^a^	266 (40)^b^
Age at index start			
Mean	48.4	47.5^b^	43.5^a^
Min-max (SD)	19.0–79.7 (14.8)	18.2–87.3 (15.7)	18.2–89.1 (15.9)
S-Creatinine at index start (μmol/l)			
Mean	62	67^a^	71^a^
Min–max (SD)	31–105 (14)	37–105 (12)	39–105 (13)
eGFR at index start (ml/min/1.73 m^2^)			
Mean	94	90^a^	88^a^
Min–max (SD)	53–136 (16)	46–127 (14)	50–122 (13)

a *p* ⩽ 0.05 compared to previous cohort.

b NS compared to previous cohort.

eGFR: estimated glomerular filtration rate; SD: standard deviation.

The treatment-related variables and renal outcomes are presented in Table 2. Time on Li was higher for Cohort 2. Compliance with monitoring guidelines improved, while S-Li concentrations decreased through the cohorts. The number of patients having Li-intoxications was highest in Cohort 1. There were only two patients who developed End Stage Renal Disease and received RRT during the study time.

**Table 2.** Treatment-related variables and renal outcomes in each cohort.

	Cohort 1 (*N* = 623), 1980–1989	Cohort 2 (*N* = 874), 1990–1999	Cohort 3 (*N* = 672), 2000–2009
Time on Li (years)			
Mean	6.2	7.1^a^	6.2^a^
Min–max (SD)	1.0–11.0 (3.3)	1.0–11.0 (3.5)	1.0–11.0 (3.4)
Compliance			
Mean (%)	75	89^a^	93^a^
Min–max (SD)	5%–100% (20%)	16%–100% (14%)	0%–100% (11%)
S-Li (mmol/l)			
Mean	0.64	0.60^a^	0.58^a^
Min–max (SD)	0.21–1.03 (0.11)	0.23–0.94 (0.10)	0.19–0.84 (0.09)
Li intoxications, (S-Li ⩾ 1.5 mmol/l)			
Patients with at least one intoxication, *n* (%)	74 (12)	72 (8)^a^	44 (7)^b^
Event = CKD4			
*n* (%)	7 (1.1)	10 (1.1)	5 (0.7)
Age at event			
Median	71.7	73.7^b^	76.1^b^
RRT			
*n* (%)	1 (0.15)	1 (0.11)	0 (0)

a *p* ⩽ 0.05 compared to previous cohort.

b NS compared to previous cohort.

CKD4: chronic kidney disease at least stage 4; RRT: renal replacement therapy; SD: standard deviation.

### Survival analysis

**Figure 2.**
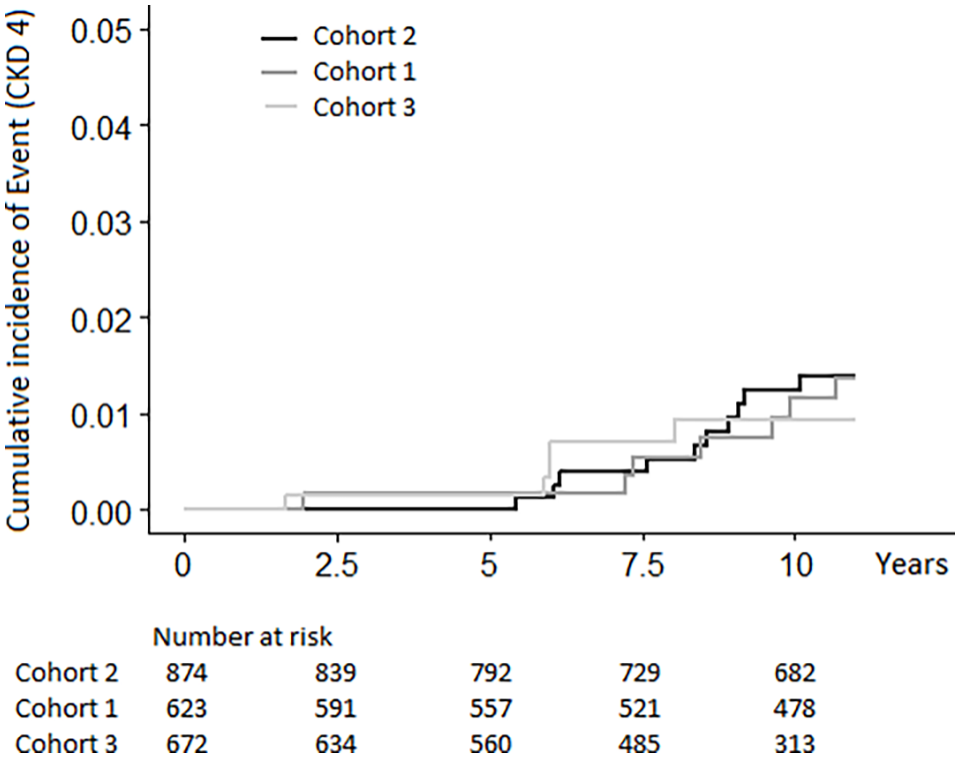
Kaplan–Meier curves showing the cumulative incidence of CKD4 for the three cohorts. CKD4: chronic kidney disease at least stage 4.

Cumulative incidence of Event is illustrated with Kaplan–Meier curves (Figure 2). Cohort 2 was selected as reference.

The Kaplan–Meier curves are fairly proportional. The mean Survival Time estimates were as follows:

- Cohort 2: 10.96 years, 95% confidence interval (CI): 10.93–10.99
- Cohort 1: 10.96 years, 95% CI: 10.92–11.00
- Cohort 3: 10.95 years, 95% CI: 10.91–11.00
- Overall: 10.96 years, 95% CI: 10.94–10.98

The Log Rank test showed no statistically significant difference in Survival Time between the three cohorts (*p* = 0.93).

Cox proportional hazards model was used to evaluate the effect of Cohort and covariates (Sex, Age at Index Start and S-Creatinine at Index Start) on survival function. The effect of Age and S-Creatinine at Index Start on Survival Time is not continuous over the whole range of values; hence, there is a need to categorise these variables. The following relevant categories were selected:

- Age at Index Start:
  ◦ Reference category: ⩽60 years;
  ◦ Category 1: >60 and ⩽70 years;
  ◦ Category 2: >70 years
- S-Creatinine at Index Start:
  ◦ Reference category: ⩽70 μmol/l for women and ⩽85 μmol/l for men (lower-medium range of the reference interval);
  ◦ Category 1: 71–90 mmol/l for women and 86–105 mmol/l for men (higher range of the reference interval).

Bivariate Cox proportional hazards models were performed, including the main variable (Cohort) and each single covariate. The covariates found to have a significant impact on survival were subsequently used in a multivariate Cox model.

Sex was not statistically significant (women vs. men: hazard ratio: 1,12, *p* = 0.80, 95% CI: 0.47–2.67) in the bivariate analysis. In contrast, Age at Index Start and S-Creatinine at Index Start were found to be statistically significant and were further included in the multivariate analysis, presented in Table 3.

**Table 3.** Multivariate Cox proportional hazards model for 2169 patients starting lithium 1980–2009.

	Regression coefficient	*p* Value	HR	95% CI for HR	
Variable				Lower	Upper
Cohort (reference: cohort 2)		0.78			
Cohort 1	−0.13	0.80	0.88	0.32	2.38
Cohort 3	−0.39	0.48	0.68	0.23	2.00
Age at index start (years) (reference category: ⩽60 years)		<0.001			
Category 1: >60–70 years	1.92	<0.001	6.82	2.38	19.52
Category 2: >70 years	2.26	<0.001	9.54	3.31	27.45
S-Creatinine at Index start (μmol/l) (reference category: ⩽70 women, ⩽85 men; category 1: >70–90 women, >85–105 men)	2.03	<0.001	7.59	3.06	18.86

CI: confidence interval; HR: hazard ratio.

Age and S-Creatinine at Index Start appeared to be important risk factors. The hazard for CKD4 was 7–10 times higher for patients who were over 60 years old at Index Start, compared to their younger peers and S-Creatinine in the higher range of the reference interval increased the hazard approximately eight times.

Even after adjusting for the covariates Age at Index Start and S-Creatinine at Index Start, the difference between the cohorts was not statistically significant. Hence, the null hypothesis is not rejected.

## Discussion

**Figure 3.**
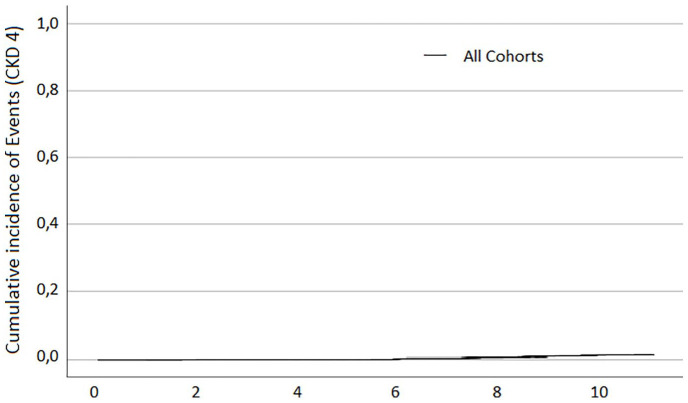
Kaplan–Meier curve showing the cumulative incidence of CKD4 for the whole study population. CKD4: chronic kidney disease at least stage 4.

The results indicate that few patients with normal creatinine at the start of long-term lithium treatment are at risk for developing severe renal damage during the first follow-up decade. In Figure 2, the *Y*-axis has been scaled down to 5% (or 0.05), to facilitate visual detection of possible differences between the three cohorts. For better visualisation of the incidence of CKD4 in the study population, a diagram with the *Y*-axis scaled up to 100% (or 1.0) is presented in Figure 3.

There was no discernible protective influence from improved compliance with management guidelines.

The assumptions underpinning the study rationale were as follows:

(a) The wide implementation of the lithium monitoring guidelines in Sweden might have contributed to optimisation of lithium exposure (aiming at the lowest effective S-Li) and fewer Li-intoxications.
(b) Important advances in the management of somatic conditions which may aggravate CKD (such as diabetes, hypertension and cardiovascular diseases) have been made during the last decades.
(c) These developments may have delayed the progression of CKD towards the more severe forms in lithium-treated patients, with or without somatic comorbidities.

The data (as shown in Table 2) confirm at least in part these assumptions, as significantly higher compliance with lithium monitoring guidelines, lower S-Li and lower proportions of patients with Li intoxications through the cohorts were noted. A post hoc analysis shows a weak, but statistically significant, negative association between mean compliance and mean S-Li (Pearson correlation coefficient −0.117, *p* < 0.001, 95% CI: −0.158 to −0.075).

However, there was no cohort effect on the hazard for CKD4 after adjusting for covariates. The renal outcome of lithium-treated patients was unchanged through the cohorts, despite differences in treatment variables.

Possible explanations for these findings are the rather high compliance (75–93%) and comparable lithium levels (0.58–0.64 mmol/l) throughout the cohorts with differences which, although statistically significant, might not have been large enough to influence renal outcome.

Another possible explanation is the relatively short follow-up time. Lithium-induced renal injury takes time to develop and in this context 10 years of follow-up might be insufficient to detect the possible differences. In a longitudinal study on 953 patients (Bocchetta et al., 2015), the development towards CKD3a and 3b was continuous over 30 years and the median time on lithium needed to reach this level of renal impairment was 25 and, respectively, 31 years. Thus, the present study cannot rule out that better treatment monitoring and optimised lithium levels might have an impact on the renal outcome in a longer time perspective.

Only 1% of patients (22 out of 2169) progressed to CKD4 in the decade following Index Treatment, and of these, two patients reached CKD4 within 2 years. Is it reasonable to ascribe a causal role to lithium in these early cases? The lithium nephrotoxic effect, although having been extensively researched, is difficult to quantify in meaningful clinical terms and still remains, to a certain extent, controversial (Clos et al., 2015; Kessing et al, 2015). The results of different studies cannot be directly compared, as both follow-up time and measures of renal impairment vary. Previous studies have focused on different renal outcomes such as decrease in eGFR and increase in S-Creatinine with treatment (Tondo et al., 2017), CKD of various grades (Castro et al., 2016; Close et al., 2014; Van Alphen et al., 2021), end-stage renal disease (Kessing et al., 2017) or renal biopsy lesions (Markowitz et al., 2000). We choose CKD4 as outcome because we considered this level of renal impairment to best match the consequences of a poorly controlled severe mood disorder, both in terms of quality of life and in terms of vital risks.

Generally, it is accepted that a small proportion of patients will develop lithium nephropathy after many years of lithium exposure. However, in a renal biopsy study, lesions characteristic for lithium nephropathy were described in patients aged 26–57, whose lithium exposure varied between 2 and 25 years (Markowitz et al., 2000). The mean lithium treatment duration in this study was 13.6 years, but renal lesions were present as early as after 2 years of lithium exposure in some cases. While it might be difficult to assess the influence of each contributing circumstance, it is conceivable that a combination of strong risk factors (cardiovascular disease, pre-existent subclinical renal injury and/or high age) may prompt an early debut of clinically manifest renal function impairment in some lithium-treated patients. A broader analysis of the various factors affecting the renal function during lithium treatment requires a longer observation time.

Besides creatinine level, age at start of lithium was a strong predictive factor for CKD4 (see Table 3). The higher risk for renal failure in elderly lithium-treated patients might be explained by the lower (and decreasing) eGFR in this age group in general, independently of lithium treatment. eGFR is known to decrease after 40–60 years of age, as part of normal ageing process. The exact age for start of decline and annual eGFR loss vary, but a thumb rule has been 50 years of age and up to 1 ml/min/1.73 m^2^. According to data published by Eriksen et al. (2020), in an adult population between 50 and 97 years old, the mean eGFR was lower with every year of age, in both women and men, both healthy and unhealthy. In this study, ‘healthy’ was defined as having no major chronic disease or risk factors for CKD. The healthy individuals had a lower yearly eGFR loss compared to the unhealthy ones (0.72 vs. 1.03 ml/min/1.73 m^2^ for men and 0.92 vs. 1.22 ml/min/1.73 m^2^ for women), but nevertheless aging overall was associated with continuous declining renal function between 50 and 97 years, in all categories.

More women than men were included in all cohorts, but their proportion was lower in the newer cohorts compared to Cohort 1. A possible explanation might be sex-differentiated prescribing pattern, as described by Karanti et al. (2015). The choice of medication for bipolar disorder in the period 2004–2011 was examined and it was found that women were more likely to be treated with antidepressants, ECT, lamotrigine, benzodiazepines and psychotherapy, while men were more likely to be treated with lithium. These findings corroborate with the launch of fluoxetine and sertraline, two of the most used serotonine reuptake inhibitors (SSRI), in the late 1980s – beginning of 1990s, and the launch of lamotrigine – in the beginning of 1990s.

## Limitations

The study is retrospective and relies on data that was not specifically collected for the study.

The low incidence of CKD4 and CKD5 over the 10 years of follow-up may lead to type 2 error. A longer timeframe may be needed to detect the possible differences.

The treatment variables were calculated based on assumptions about laboratory data; one year without any lithium measurement was regarded as treatment interruption, but in reality, it could be a treatment period with very poor compliance.

Three variables were used in the multivariate analysis (Cohort, Age at Index Start and S-Creatinine at Index Start), while the total number of events was 22. This gives less than 10 events per variable (EPV), in breach with the rule of thumb stating that Cox model shall include at least 10 EPV. However, Vittinghoff et al. (2007) have shown that the model's performance when using 5–9 EPV is fairly comparable with that for 10–16 EPV, especially when the multivariate model is used to address adjusting for covariates.

No adjustments have been made for somatic comorbidities that play a role in the progression of renal impairment, nor for associated medications. This is an important limitation, as later cohorts may have included a higher proportion of patients with somatic comorbidities. An indication of this might be the lower eGFR at Index start in the later cohorts, despite a lower Age at Index Start.

## Conclusions

The risk for severe renal damage is low, but may occur during the first decade of lithium treatment. Our data suggest that improved compliance with guidelines for monitoring of long-term lithium treatment is not reflected in less risk for severe renal damage. Other factors appear to be of greater significance. Longer observation time would be needed to detect the possible differences.
